# The Surgeon

**Published:** 1861-11

**Authors:** E. Andrews


ARTICLE III.

### THE SURGEON:

AN INTRODUCTORY LECTURE, DELIVERED BEFORE THE MEDICAL DEPARTMENT OF LIND UNIVERSITY, SESSION 1861-2. OCTOBER 14, 1861.

By Prof. E. ANDREWS, A. M., M. D.

*Published by Request of the Students.*

GENTLEMEN :—We live in an hour, such as comes to men but once in a lifetime. Medical science and art, which have hitherto illumined the hours of peace, are summoned to the battle-field to front the grim realities of war; and not only our profession, but all others are mingled in a struggle which moulds the characters of millions, and which will stamp the impress of the coming century.

At such a time, I cannot talk to you of common petty interests apart from the influence of the great convulsion. It is War that speaks, not I. I must talk to you as men to be moulded by the influence of this tremendous hour, for the destinies of another age than that which has just past.

*War is a moral tonic.* It comes to the social world, as the whirlwind and the earthquake come to the natural. It finds many flimsy, social fabrics in its path, erected for the time of peace. They are cheaply glorious with paint, and gorgeous with stucco, but the sweep of the tempest bears them away like chaff in its passage, until that which is truly strong and giant-like, alone remains.

In times of long peace, men lose their hold on strong first principles. They plan flimsy and insecure schemes of business; their social fabrics are artificially painted, and stuccoed and bedizened with nonsensical observances. Education grows out into distorted and strange forms, and even the learned professions spin theories frailer than cobwebs, and take to forming artificial rules of conduct, and false notions of their relations to the rest of the community.

So then, I, for one, rejoice that this war has come. Not only *our* profession, but others, and the social and civil organizations themselves, have grown up too rank and luxuriant for safety. They need the tossing of the blast to harden their limbs, and the shock of war to consolidate their strength.

I know that the evils of war are great. There is paleness on many brows. For the first time the nation is shaken by a real danger, and sees flapping over her stars, the wings of a tempest whose thunders mutter of final dissolution. But in hours like this, God renovates communities, and purges away their errors, their luxury and their corruption. It is with throes like these that heroes are born—amid convulsions such as these, nations grow giant-like, and renew their vigor, their youth and power.

Americans had come to fancy that all their institutions were to be established forever, without a struggle. They verily thought that the duty of defending their country was one which could never be practically exercised. Even our civil freedom, since the throes of her birth, has not been put to serious peril. Her infancy was cradled among the green valleys of the east; in childhood she sported securely on the shores of the great lakes; and on the prairies of the west, we have watched with pride the rounding cheek of her youth; but now her majority is attained, the hour of her bridal draws near; this day freedom weds with the sword, and pours out on the battle-field, the red wine of her marriage feast.

In times like these, there are no sluggards: the very air is full of electric life, and honest vigor of action becomes a luxury. Let us, therefore, who are not called to the battle-field, where some of us may lay our bones before another year, let us take a new view of our privileges, responsibilities and duties with the truthful vision imparted by this auspicious hour.

If I am equal to the occasion and the theme, I wish to portray to you to-night, the character of the *True Surgeon* for the present and the coming time.

First, then:—What is demanded to constitute a good surgeon? I answer,—the primary requisite for a good surgeon, is *to be a man*,—a man of courage, a man of moral rectitude, a man of broad views and noble sentiments. Narrow-minded, mean-souled, little-brained men, ought never to plunge into the learned professions. Their circumstances bewilder them; they become entangled in their learning like a fly in a cobweb; their language is barbarized by technical phrases; their mental vision is obscured by professional squints, and their hearts, cut off from the great life-current of humanity, dry up to nonentities. Such men never grasp the grand and inspiring truths at the core of their profession, but flounder bewildered among its accidental surroundings. Hence, we have lawyers, learned in statutes and decisions, and reckless of justice; clergymen, deep in theology, and devoid of religion; and surgeons, skilled in anatomy and profoundly stuffed with technicalities, but perverted in judgment, rotten in morals, and destitute of sympathy. In one word, they are *not men*; they are only the dried-up shells of preachers, lawyers and doctors, with all the manhood blown out of them.

Among the particular traits which go to make up a manly character, one of the first, and one which is eminently necessary to a good surgeon, is courage. In battle, a portion of the surgeons go with the line, and dress the wounded under fire. In the Crimea, the British surgeons dressed the wounded in the trenches where they fell; and the results, both in their wars and our own, show that bullets have no natural repugnance to killing medical officers. Unless, therefore, a man can tie up an artery with a cool head and steady hand, while the bullets are singing past him, he will not do for a surgeon. Again, he must sometimes walk his hospital day by day, when it is filled with contagious and pestilential disease, breathing an air more deadly than that of the battle field. If he cannot do this without flinching, he will not do for a surgeon.

And again, in civil practice there are cases which quick, skilful, and daring action alone can save; an instant only is given for a human life, and the surgeon who is aghast and hesitates at the emergency, loses his patient. I tell you, gentlemen, these moments will test your courage more severely than bullets whistling across the line of battle.

Another requisite of high professional quality, is breadth of knowledge, not only of the practical branches, but of those general and preliminary topics which are necessary to constitute a well-educated man. The intensely practical character of the American mind has unfortunately misled many students as to the real requisites of high success. Every year, medical teachers receive applications from men who desire the honors of a diploma, but wish to be excused from attention to, what they call, the non-practical branches. They imagine that but little more is necessary for a physician than theory and practice, materia medica, and obstetrics; and for a surgeon, than anatomy, surgery, and a case of instruments. One man who claimed to be eminent already in surgery, objected to pathology as not practical; it was not necessary to study the nature of the disease, the practical thing was to cure it. God help his patients; I think they had better send for a butcher, as being the more practical man of the two.

Gentlemen—true eminence is not built on such small foundations. The old kings of Egypt erected many vast pyramids, which have defied the assaults of time, but none of them were built with the small end downward. Make broad the foundations, and then build on them with a liberal hand. There is a great error prevalent as to what is really practical; an error which has consigned to hopeless inferiority, many a capable young man. Common sense, which is another name for a well-balanced mind, is essential in all professions; hence, everything is practical to a surgeon which gives him breadth of thought, evenness of judgment, and influence in the community.

The fact that some men have achieved notoriety on what, at first glance, seems a very narrow basis, is no contradiction of my position. A closer examination of the characters of these men, show that they are all of two classes. First, men who have blown up a kind of balloon-greatness by a special talent for imposing upon the public; and secondly, men who have made good their early deficiency by industry and superior natural capacity for gathering knowledge. The latter will always be found to have acquired in this way, a broad foundation before they attain any substantial eminence.

There is no doubt, whatever, that if circumstances permitted, it would be better for every young man to take a four years' college course, before commencing his proper medical studies, though at present, such an extensive preliminary course is out of the reach of many, who may yet become superior surgeons and physicians. But fortunately, knowledge is power, however obtained, and the man, who, by industry will acquire it, though it be by hours and moments, stolen from other occupations, may reap its full fruits. Now, gentlemen, the way to commence is very plain. It is perfectly easy to point out a course, by which a young physician, even of deficient preliminary education, can become the recognized superior over most of his fellows of equal natural talent. He must, like Elihu Burrit, the learned blacksmith, make up by after study, his lack of early advantages.

The first years of a practitioner's life are not usually fully occupied with business. Of the leisure hours on hand, let one-half be devoted, for six years, to general literature and science, and the other half to reading, writing, and hard thinking directly upon his profession. I guarantee to any young practitioner of fair capacity, who will do this faithfully, a superiority over his fellows, which will be publicly known and recognized during the rest of his lifetime. A good education necessarily implies a variety of knowledge. Each kind of study leaves only its own special mark on the mind, and hence, acquisitions of several kinds are necessary to a full development. The languages are of constant use in prescriptions, and in technical terms; but besides this, the exercise of translating gives a man ease of grasping thought, and facility in expressing it. Mathematics confer precision of reasoning, and accuracy in mechanical contrivance, which a surgeon especially requires; while natural sciences give breadth and scope of thought. I earnestly urge a liberal knowledge of these various branches. But beware of becoming so entangled in the meshes of your studies, that they become master of you, and not you of them. A few men become so fascinated with these pursuits as to follow them to the exclusion of all professional improvement, and to the loss of public confidence in business. You should pursue them not as an end, but as a means, in order that by self-culture, you may bring to your professional duties a finer and more powerful mind—that you may become more perfect men, more splendid surgeons.

Some physicians have become so enamored of literature, as to actually forsake their profession for it. Some have studied mathematics and forgotten physic, and others have followed metaphysics until they have lost sight of earth altogether.

There is a class of men who become so absorbed in natural science, as to forget and neglect their proper surgical and medical studies. Endowed with unusually sensitive minds, their contact with nature in her magnificent solitudes, begets a joy that abstracts them from the more puny operations of art. The mental effect of this error is peculiar. A man may contemplate the beauty of mountain cliffs, of misty valleys, of stars and rosy sunsets, until he undervalues by comparison all other things. He may wander, so to speak, in the secluded valleys of science, where the south-wind rustles warm in the foliage, where the streams babble, and the wild vines trail their purple clusters, until like Actæon rashly beholding the beauty of Diana, his nature is changed and loses its manhood. He becomes too shrinking and sensitive to deal with the disease, ugliness, and depravity of man. In fact, he has reposed so long amidst the beauty of the works of God, that he is no longer fit to come out and do battle with the world, the flesh and the devil. It is true such men are very estimable, but they are not practical. Contact with the world stings them sorely, and they retire constantly to the shelter of their favorite studies, until at length the world says they are very learned, but too fussy, and too much devoted to birds, insects, plants and snakes to attend properly to the wants of the sick. Study natural sciences therefore, not to an inordinate excess, but just enough to cultivate that breadth of thought and gentleness of heart required by a true and noble surgeon. Other branches of general learning require similar cautions. You are not to become eminent in them, but you must make use of them, to render your intellect quick and bright, and kindly and strong in your professional duties.

One thing which is necessary to a sound and reliable surgeon, is an integrity beyond the reach of selfishness, and a sense of honor above the possibility of stain. There are occasions in surgery, as in all other arts, when sacrifice of principle may yield a temporary advantage. A tumor may be excised to the great astonishment and admiration of the beholder, but not always to the advantage of the patient. A limb may be amputated for a large fee and great eclât, when to save it would yield less pay and no renown, for the extra labor involved.

I am sorry to say that there are men who allow their judgments to be warped by these considerations, and who will even act with entire recklessness of a patient's welfare, for the sake of a brilliant surgical operation. In short, they are scoundrels, and men who put themselves under their charge, fare as men do, the world over, who trust their interests in scoundrels' hands.

Such surgeons are no common villians. Other men may have palliations for their crimes. The thief may be in want, the ambitious man only desires power, whose exercise may be correct, and even the revenger flatters himself that he is exercising a species of justice; but for him, who has dipped his hand unnecessarily in living blood, who shall seek an extenuation. His heart is no longer that of a man, but of a hyena, that cannot wait for the dead to glut its desire. There can be no more pitiable sight than an old surgeon, at the end of his days, who has lived such a life. He has spent his time amidst horrors and bloodshed, without a single noble incentive to soften the ugliness of the retrospect, and we may well believe that the memory of the murdered will haunt his dying pillow. But of you, we expect better things,—things worthy of this inspiring era, when the very air is full of the cry, *Quit yourselves like men!* Let your memories be those of a surgeon who has done his whole duty. He has saved the shattered limb, and restored it to vigor; he has cleared the vision of the blind; he has laid his hand where the crimson blood was spouting out the life, and stayed its flow. He has insulted Death in his own dominions, and flung the monster off, after he had fastened on the very throat of his victim. Life is longer, and humanity is happier for such a man's existence.

Since the very object of our profession is to relieve suffering, it may seem strange that any advice should be offered on the subject of humanity; but just as the almoners of public charity, by constant contact with pecuniary distress, sometimes become the most hard-hearted towards the poor, so surgeons may become callous, by the constant presence of suffering, until they are not only cold and heartless in manner, but actually brutal and reckless of pain in their operations. It is to be regretted that some teachers of surgery, now dead, but who were men of talent while they lived, have exerted a most pernicious influence in this regard. It was said by the old Latins, "*nil de mortuis, nisi bonum*,"—nothing but good should be spoken of the dead; but I prefer the still older maxim of the Egyptians, that "the dead must abide the judgment of the living." I repeat, therefore, that some men of excellent attainments and great mental power, have, during their lifetime, set an example of brutal roughness of manner, which has had a most disastrous effect upon their disciples. Young men impressed with the splendid skill and commanding talents of these instructors, have naturally imitated their faults, fully as much as their virtues. Accordingly, there are in almost every part of the United States, some practitioners, who, to this day, believe that a domineering harshness of manner, set off on great occasions by a few paragraphs of profanity, are the genuine marks of a superior surgeon. Nothing can be more erroneous. The ancient ideal of a true knight, is just about the real model for a surgeon. A courage that never wavered in front of danger, and a heart that was never cold to distress,—kind, courteous and brave. Of the famous Chevalier Bayard, the praise was, "*sans peur et sans reproche.*"—without fear and without reproach. Another bold warrior, after his death, was thus lamented,—"And thou wert the kindest man that ever put spear in rest." An epitaph, that could hardly be put on the tombstones of some of our departed surgeons. I am well aware that a surgeon should be calm. His sympathy should not boil over into a hysterical excitement; it *must not disorder the lightest motion of his hand;* but he must carry in his heart, that abiding kindness which will insure him against exciting a single useless terror in the patient's mind, or a single needless pang of bodily pain.

Among the qualities of a true surgeon, not the least important is his enthusiasm, for on this depends his enjoyment, and consequently, his diligence in his work. You occasionally hear men say, that the *study* of medicine and surgery is very interesting, but the practice is tedious and dull. Now I can assure you, that to a well-trained mind, this is an unmitigated lie. Practice is dull, only to those who do not think, reason, and study about their cases; in short, who are routine practitioners, not acting from the deductions of a living reason, but raking forever among the ashes of a dusty memory. Make your practice a perpetual study, and it will be a perpetual enjoyment. To him who attentively thinks of all his cases, the truths of nature unfold themselves in wider and wider fields. Every day you reach a higher stand-point, and a broader view, every week you master some practical difficulty, and every year you learn to conquer some bodily evil, which before defied your efforts. Progress, like this, will make any man enthusiastic, and the enthusiastic man is happy.

But if a man considers his education complete, when the seal is applied to his diploma,—if, instead of continuing to develop, he merely subsides into a mechanical way of taxing his memory for rules of practice, he will certainly not find it agreeable. There is a class of such physicians who grow worse as they grow older. Their very experience is a danger to them!—they learn nothing by it, and in fact, are never as good practitioners as when they first graduated.

Gentlemen,—set it down as certain, that there is something wrong about an old physician who thinks that practice is not interesting. Among well-balanced minds, there are, however, some which naturally give preference to medicine, and others to surgery; so that to a certain extent, this division of the profession into two parts, marks two classes of minds. This preference depends on differences of the two positions, and the relative mental qualities brought in play. Thus medicine requires more breadth and philosophical depth of thought than surgery, but less precision and mechanical ingenuity; and accordingly, minds seek the sphere which best indulges their favorite bent. There is a difference, too, in the social relations of the two classes. A good physician secures a warm, earnest attachment from his patrons. There is a circle of families who look on him as a permanent and confidential friend, and whose attachment is not valued by him at its mere product in dollars and cents. A man of fine sensibility cannot but highly prize this steady and earnest regard, which is the reward, peculiarly, of medical skill, and moved by its stimulus, he will naturally engross himself, more and more, in that kind of practice. The surgeon, on the other hand, has a less warm but a more glittering reputation presented to his ambition. His patrons are transient. They come, perhaps, hundreds of miles for a single operation, and being relieved, return to their homes, and disappear from the circle of his acquaintance. Of course, they cannot stand in the relation of intimate daily friends. To balance this, they look upon an eminent surgeon much as they do upon a renowned soldier. They yield the tribute of high admiration to one who dares to put his hand to the machinery of life, and can, with impunity, take out its living wheels. If a surgeon's renown is less warm than a physician's, it is wider and more splendid.

As might naturally be expected, surgeons and physicians, considered as classes, have each their peculiar faults and virtues. As surgical appliances are less complex, and more easily understood than medical, so surgeons, on the average, are more clear and accurate in their ideas than physicians, and less in danger of running away into obscure theories, which neither are, nor can be definitely proved. Even quacks are obliged to keep somewhere near the truth, when they meddle with surgery. But on the other hand, these very advantages draw many inferior men to the surgical ranks. As surgery is easier to comprehend, too many of its votaries are superficial and narrow—not possessed of sufficient philosophical power to grasp easily the truth of medicine. As its renown is glittering and brilliant, it attracts men, too often, who are more cold and selfish, than physicians—men incapable of warm friendships, over eager for notoriety and greedy of gain. So it has been in time past. I call upon you to take this stain away from the profession, by bringing to it hearts full of generous emotions; hearts whose rich pulses of youth live on in riper age; hearts that love nobleness and despise meanness, and that feel for the sufferings of the afflicted.

The difference of mechanical talent in different men, is a reason, also, why they love this art in unequal degrees. A first-rate surgeon must be a natural mechanic. This trait depends mainly upon the clear conception and easy memory of forms, localities and distances. To him that possesses it, the human frame is transparent, with all its nerves and vessels; his mind's eye sees through the clotting blood of wounds, to the vessels that yield the stream, and his unerring finger makes out with mathematical certainty, the nature of injuries, which others fumble over in vain for a solution. To such a man the human frame is plastic, and is moulded to his wishes; while to another, it is full of dangerous accidents to alarm the unwary operator.

Mechanical ingenuity is also necessary in devising, changing, and applying instruments. A million of dollars will not buy sufficient instruments for a man who has no ingenuity, while he who has this talent, has an armament in his brains sufficient for every purpose, and which will get him out of every difficulty. One man will amputate a thigh, creditably, with only a penknife and a gimlet, while another will bungle the operation, though he have an instrument-factory full of knives and saws.

The last and greatest qualities which I mention for the surgeon, are *knowledge of the art, and industry for its improvement.* Let no man touch its threshold who is not determined to add to its resources by discoveries of his own. We have already made great advancement, since the day when chloroform was unknown, when smooth cuts were stuffed with lint, and stumps, after amputation, had to be dipped in hot pitch, to stop the blood; but still greater things await us in the future, and he that seizes on new truth, and embodies it in practice for the benefit of man, is doing service for all time.

The history of the past is finished and sealed up; its virtues and its errors were great, but let the dead bury their dead. To-day, God opens a new volume in the nation's history, whose very first page is blazoned with war and carnage, and calls for men of sterner and of loftier mould than before. Come up to the great work of the age, and do your part like men, and the results will place you among the world's benefactors.

I believe in the onward destiny of the human race, and I desire that our profession shall play an honorable part in the world's increasing progress. Make yourselves, therefore, superior to the fathers, and be ye ready for the coming hour. Heaven rules over the convulsions of nations, and works from each, a higher destiny than before. After a time, peace will return, ladened with worthy fruits, and when at length the destined changes are wrought out, and the gorgeous fabric of a new civilization shall be complete, no shadow dimming the splendor of its halls, the united nation will rejoice in it, and God, looking upon his work, once more pronounce it very good!

